# A phase I clinical trial for [^131^I]meta-iodobenzylguanidine therapy in patients with refractory pheochromocytoma and paraganglioma

**DOI:** 10.1038/s41598-019-43880-6

**Published:** 2019-05-20

**Authors:** Hiroshi Wakabayashi, Anri Inaki, Kenichi Yoshimura, Toshinori Murayama, Yasuhito Imai, Tetsuya Higuchi, Megumi Jinguji, Tohru Shiga, Seigo Kinuya

**Affiliations:** 1 Department of Nuclear Medicine, Kanazawa University Hospital, Ishikawa, Japan; 2 Department of Biostatistics, Innovative Clinical Research Center, Kanazawa University Hospital, Ishikawa, Japan; 3 Department of Clinical Development, Kanazawa University Hospital, Ishikawa, Japan; 4 Department of Data Center, Innovative Clinical Research Center, Kanazawa University Hospital, Ishikawa, Japan; 5 Department of Diagnostic Radiology and Nuclear Medicine, Gunma University Graduate School of Medicine, Gunma, Japan; 6 Department of Radiology, Kagoshima University Graduate School of Medical and Dental Sciences, Kagoshima, Japan; 7 Department of Nuclear Medicine, Hokkaido University, Hokkaido, Japan

**Keywords:** Phase I trials, Adrenal tumours

## Abstract

Refractory pheochromocytoma and paraganglioma (PPGL) have a poor prognosis and the treatment strategy remains to be established. This multi-institutional phase I study was performed to determine the safety, dose-limiting toxicity (DLT), and efficacy of [^131^I]-meta-iodobenzylguanidine (^131^I-mIBG) therapy for refractory PPGLs. Twenty patients with refractory PPGL were enrolled in this study. We administered fixed doses of ^131^I-mIBG to all patients, delivering a second and third course of ^131^I-mIBG to eight and three patients, respectively. During the 20 weeks after ^131^I-mIBG injection, the authors surveyed the adverse events in accordance with the Common Terminology Criteria for Adverse Events. All patients experienced adverse events and adverse reactions, but none experienced a grade 4 adverse event. Twelve weeks after ^131^I-mIBG injection, examinations for the evaluation of therapeutic effects was performed in accordance with the Response Evaluation Criteria in Solid Tumours (RECIST). The best overall response rates (based on RECIST categories) were 10% (complete response), 65% (stable disease), 15% (progressive disease), and 10% (not all evaluated). The efficacy and safety of ^131^I-mIBG therapy was shown in patients with refractory PPGL, and DLT was observed in neither single nor repeated ^131^I-mIBG therapy, indicating a tolerability for ^131^I-mIBG therapy.

## Introduction

Since the 1980s, patients with unresectable, metastatic, and relapsed pheochromocytoma and paraganglioma (refractory PPGL) have been widely treated with radioisotope [^131^I]meta-iodobenzylguanidine (^131^I-mIBG) radiotherapy to cure or control inoperable tumors^1^. Through active uptake via norepinephrine transporter or passive diffusion, the mIBG radioisotope, a guanethidine analog resembling norepinephrine, can enter chromaffin cells where it is stored in catecholamine-containing neurosecretory granules, leading to the anticancer effect of ^131^I-mIBG therapy on lesions^2–5^.

In combination with surgery, external radiotherapy in the form of ^131^I-mIBG therapy has been performed to eradicate tumors. PPGLs occur in 2–8 per million persons per year and refractory PPGLs are very rare, occurring in 10–30% of PPGL cases^6–8^. Although ^131^I-mIBG therapy has been in use for over 30 years, researchers have conducted only a few phase clinical trials in patients with refractory PPGL regarding its safety and efficacy. Two phase II trials of high-dose ^131^I-mIBG therapy requiring preparation of autologous hematopoietic stem cell rescue for the severe adverse effect of bone marrow suppression^9,10^ and one phase I trial of a high-specific activity of ^131^I-mIBG therapy^11^ exist. No other phase trial confirming the safety and efficacy of ^131^I-mIBG therapy has been conducted^12^. A wide range of response rates to ^131^I-mIBG therapy on imaging (0–83%) and in hormonal examinations (20–100%) in patients with refractory PPGL was reported in a meta-analysis of retrospective studies^13^.

This phase I trial in a multi-center setting was conducted to assess the safety, dose-limiting toxicity (DLT), and efficacy of ^131^I-mIBG therapy in patients with refractory PPGL.

## Results

We enrolled 20 patients between February 2016 and July 2017 (Table 1). After the patients’ initial diagnoses of PPGLs refractory to therapy, 5.14 ± 4.90 years (range: 0.3–17.8 years) had passed. We described refractory PPGLs with severe local invasion at initial diagnosis (25%, 5/20), metastasis at initial diagnosis (25%, 5/20), local recurrence after surgical resection (20%, 4/20), and metastasis after surgical resection (65%, 13/20).

**Table 1:** Characteristics of patients.

Sex (M:F)	14:6
Age	51.2 ± 14.4 (range: 21–76) years
Diagnosis	
Pheochromocytoma:paraganglioma	13:7
Complications	18 (90.0%)
Hypertension	11 (55.0%)
Tachycardia	1 (5.0%)
Arrhythmia	2 (10.0%)
Orthostatic hypotension	1 (5.0%)
Headache	3 (15.0%)
Palpitation	1 (5.0%)
Cold sense	1 (5.0%)
Constipation	4 (20.0%)
Symptom	
Headache	2 (10.0%)
Epigastric pain	1 (5%)
Anginal pain	1 (5%)
Palpitation	2 (10.0%)
Frigidity	2 (10.0%)
Nausea and vomiting	5 (25.0%)
Constipation	5 (25.0%)
Laboratory examinations (range, outside of normal range (%))	
White blood cell (*10^3^/mm^3^)	5.5 ± 1.6 (range: 3.0–9.6, 10.0%)
Platelets (**10^4^/mm^3^)	20.3 ± 6.7 (range: 12.9–38.5, 20.0%)
Hemoglobin (g/dL)	13.4 ± 1.5 (range: 11.0–16.6, 30.0%)
eGFR (mL/min/1.73 m^2^)	76.4 ± 21.3 (range: 46.4–131.87, 30.0%)
HbA1c (%)	5.7 ± 0.7 (range: 5.1–7.8, 15.0%)
BNP (pg/mL)	21.6 ± 35.8 (range: 4.0–165.9, 35.0%)
Plasma adrenaline (ng/mL)	1.5 ± 3.3 (range: 0.005–12.0, 25.0%)
Plasma noradrenaline (ng/mL)	76.4 ± 168.1 (range: 0.1–563.0, 55.0%)
Plasma dopamine (ng/mL)	2.0 ± 4.4 (range: 0.005–14.0, 35.0%)
Urinary adrenaline (μg/day)	16.3 ± 36.8 (range: 0.5–163.2, 15.0%)
Urinary noradrenaline (μg/day)	545.3 ± 1162.6(range: 87.6–5310.0, 75.0%)
Urinary dopamine (μg/day)	1311.5 ± 936.7(range: 460.0–4000.0, 45.0%)
Urinary metanephrine (mg/day)	0.4 ± 1.0 (range: 0.01–4.62, 35.0%)
Urinary normetanephrine (mg/day)	6.8 ± 14.6 (range: 0.15–52.0, 80.0%)
Urinary vanilylmandelic acid (mg/day)	16.0 ± 31.3 (range: 3.1–139.0, 55.0%)
Urinary homovanillic acid (mg/day)	4.5 ± 1.4 (range: 2.7–9.5, 10.0%)

eGFR: estimated glomerular filtration rate; HbA1c: hemoglobin A1c; BNP: brain natriuretic peptide.

We administered ^131^I-mIBG therapy at doses of 7.4 and 5.55 GBq to patients at first treatment in this study. All patients were evaluated for response assessment. We performed a second and third course of ^131^I-mIBG therapy in eight (40%) and three (15%) patients, respectively. We treated two patients following our protocol (10%). However, the protocol was stopped in 18 patients (90%) as the attending physicians decided on discontinuation (12/20, 60%), based on PD in scintigraphic response (2/20, 10%), PD in best overall response based on Response Evaluation Criteria in Solid Tumors (RECIST) (1/20, 5%), PD in both scintigraphic response and overall response based on RECIST (1/20, 5%), and proposed discontinuation of therapy (not related to adverse events) (2/20, 10%).

### Safety evaluation

We found no DLT in any patient. Although all patients had adverse events and adverse reactions, there was no death event within 6 months after enrollment. However, one patient died due to disease progression during the study period (5%).

The adverse events and reactions occurring at any grade in ≥50% of patients were in Table 2. The observed rate of adverse events and reactions did not differ between each course of ^131^I-mIBG therapy. We encountered no grade 4 adverse event in this study. We observed no unexpected changes in investigations, vital signs, and physical signs.

**Table 2:** Safety evaluation of ^131^I-mIBG therapy.

Adverse events by SOC	
Investigations	19/20, 95.0%
Gastrointestinal disorders	17/20, 85.0%
Metabolism and nutrition disorders	14/20, 70.0%
General disorders and administration site conditions	10/20, 50.0%
Adverse events by PT	
Thrombocytopenia	15/20, 75.0%
Loss of appetite	14/20, 70.0%
Lymphopenia	13/20, 65.0%
Nausea	11/20, 55.0%
Leukopenia	10/20, 50.0%
Adverse reactions by PT	
Thrombocytopenia	15/20, 75.0%
Loss of appetite	14/20, 70.0%
Lymphopenia	13/20, 65.0%
Nausea	10/20, 50.0%
Leukopenia	10/20, 50.0%

SOC: system organ classification, PT: preferred term.

### Response evaluation

The best overall response rate based on RECIST was 10% (2/20) in complete response (CR), 65% (13/20) in stable disease (SD), 15% (3/20) in progressive disease (PD), and 10% (2/20) in not evaluated (NE). The response rate [partial response (PR) + CR] was 10% [95% confidential interval (CI): 1.2–31.7%] (Table 3).

**Table 3:** Response evaluation.

No	Registration data	Refractory Pheochromocytoma/Paraganglioma	Response to ^131^I-mIBG therapy			
			BOR based on RECIST	CE based on ^123^I-mIBG scintigraphy		
				1^st^ course	2^nd^ course	3^rd^ course
1	2.29.2016	Pheochromocytoma	SD	CR	—	—
2	3.4.2016	Pheochromocytoma	SD	PR	PR	PR
3	3.17.2016	Paraganglioma	SD	PR	SD	PR
4	5.11.2016	Pheochromocytoma	NE	non-CR/non-PD	PD	—
5	5.18.2016	Paraganglioma	SD	PR	—	—
6	6.9.2016	Paraganglioma	PD	PD	—	—
7	7.13.2016	Pheochromocytoma	CR	CR	CR	—
8	7.29.2016	Paraganglioma	SD	SD	SD	—
9	7.29.2016	Pheochromocytoma	SD	SD	SD	PR
10	10.6.2016	Pheochromocytoma	PD	PD	—	—
11	10.13.2016	Paraganglioma	PD	PD	—	—
12	10.24.2016	Paraganglioma	SD	PD	—	—
13	11.24.2016	Pheochromocytoma	CR	PR	PR	—
14	1.13.2017	Pheochromocytoma	SD	SD	SD	—
15	2.6.2017	Pheochromocytoma	SD	SD	—	—
16	2.9.2017	Pheochromocytoma	SD	PR	—	—
17	2.10.2017	Pheochromocytoma	SD	SD	—	—
18	3.1.2017	Pheochromocytoma	NE	SD	—	—
19	4.21.2017	Pheochromocytoma	SD	SD	—	—
20	7.14.2017	Paraganglioma	SD	SD	—	—

mIBG: meta-iodobenzylguanidine; BOR: best overall response; CE: comprehensive evaluation; RECIST: Response Evaluation Criteria in Solid Tumours; CR: complete response; PR: partial response; NE: not evaluated; PD: progression disease; SD: stable disease.

The scintigraphic response in the first course was 10% (2/20) in CR, 25% (5/20) in PR, 40% in SD (8/20), 20% in PD (4/20), and 5% in non-CR/non-PD (1/20). Response rate (PR + CR) was 35% (95% CI: 15.4–59.2%).

Eight patients received a second course of ^131^I-mIBG therapy. The scintigraphic response in the second course was 5% in CR (1/20), 10% in PR (2/20), 20% in SD (4/20), 5% in PD (1/20), and 60% in unknown (12/20). The response rate was 15% (95% CI: 3.2–37.9%).

Three patients received a third course of ^131^I-mIBG therapy. There were three patients with PR (15%) and 17 patients with unknown (85%) in the scintigraphic response. The response rate was 15% (95% CI: 3.2–37.9%).

There was no death within 6 months of enrollment and overall survival (OS) rate was 100%. We did not determine the median OS statistically due to no death event. Four patients had progression events during the 6 months since enrollment and progression-free survival (PFS) at 6 months was 80.0%. In the follow-up period, five patients had disease progression and we did not determine median PFS statistically because we observed too few events.

## Discussion

In this phase I multi-institutional clinical trial, along with the standardized treatment protocol, we evaluated the safety and efficacy of ^131^I-mIBG therapy for 20 patients with refractory PPGLs. We observed no DLT, defined as grade ≥ 4 hematological toxicity and grade ≥ 3 non-hematological toxicity, in our trial. There are no reports of prospective trials for the evaluation of adverse effects and reactions and our data agreed with previous reports of toxicities. Although selection bias might have occurred in retrospective cohort studies—especially for rare diseases—those studies revealed that the incidence of grade 3 or higher adverse reactions was quite low and grade 4 hematological toxicity had never occurred at the fixed dose of 7.4 GBq of ^131^I-mIBG^14–16^. Herein, we showed the tolerance without DLT of ^131^I-mIBG therapy.

The response rate (CR + PR) in our trial was 10.0% (95% CI: 1.2–31.7%) based on RECIST and 35% (95% CI: 15.4–59.2%) based on ^123^I-mIBG scintigraphy. These are comparable with those obtained in a review of 116 patients with malignant PPGLs^14^. The initial response rate (CR + PR) in the review of radiological abnormality and diagnostic ^131^I-mIBG scintigraphy was 30% of the patients. Based on both our data and previous reports, we consider that a response rate for ^131^I-mIBG therapy based on imaging modalities would be approximately 10–30%.

We were able to safely administer 7.4 GBq of ^131^I-mIBG therapy two or three times with no difference in the rate of adverse events compared with that of the first course of ^131^I-mIBG therapy (mean cumulative ^131^I-mIBG therapy dose: 11.4 ± 5.7 GBq). A previous review reported the repeated ^131^I-mIBG therapy with a mean single therapy dose of 5.8 GBq; the number of times the ^131^I-mIBG therapy was repeated ranged from 1 to 11 (mean: 3.3 ± 2.2 times), and the cumulative^131^I-mIBG therapy dose was 3.6–85.9 GBq (mean: 18.1 ± 13.0 GBq)^14^. The common total administered activity of repeated ^131^I-mIBG varied between 10 and 40 GBq^17^. In a Japanese multi-center observation study, 31% of patients received repeated ^131^I-mIBG therapy of individual doses ranging from 3.7 to 14.8 GBq without severe adverse effects^15^. Our trial and these previous reports support the claim that the adverse effects of repeated ^131^I-mIBG therapy are tolerable.

Systemic treatment regimens for refractory PPGLs have not yet been standardized^18^, and the optimal timing for starting ^131^I-mIBG therapy remains under investigation. We confirmed here the safety and favorable tolerance of ^131^I-mIBG therapy. There are some arguments for promoting the use of ^131^I-mIBG therapy at an earlier stage: (1) due to disease progression, including tumor cell dedifferentiation, loss of specific neurotransmitter transporters, and gene expression, mIBG does not accumulate in parts of PPGL lesions^19–24^; and (2) sometimes, refractory PPGLs rapidly progress within a few months or years after initial diagnosis. Of course we should consider that some patients could live for >10 years in a status of stabilization^25,26^ regardless of the 5-year survival rate of 40–50%^19,27,28^. However, due to the demonstrated safety and tolerance of ^131^I-mIBG therapy, our trial would support the early induction of ^131^I-mIBG therapy after the initial diagnosis of refractory PPGLs.

There are few limitations of this study. First, follow-up period was insufficient to monitor long-term side effects; patients need to be carefully followed for hypothyroidism and a second occurrence of malignancy. Second, we did not evaluate improvements in catecholamine values and clinical symptoms as endpoints. Third, the number of patients was small. This limitation was difficult to overcome, given the relatively few patients with refractory pheochromocytoma who met the inclusion criteria. Thus, repeated ^131^I-mIBG therapy needs to be investigated in a larger, multicentric prospective study.

## Conclusions

We showed the safety and efficacy of ^131^I-mIBG therapy in patients with refractory PPGLs during 20 weeks after the injection, indicating that ^131^I-mIBG therapy has a tolerability. We observed no case of grade 4 toxicity in repeated ^131^I-mIBG therapy. In addition, repeated ^131^I-mIBG therapy could be performed without severe adverse events in patients with refractory PPGLs.

## Methods

### Study outline

Following the previously published study protocol^12^, we enrolled 20 patients with refractory PPGLs in this study. We screened the patients in accordance with both inclusion and exclusion criteria and gave the registered patients fixed doses of 5.55 GBq or 7.40 GBq of ^131^I-mIBG. We surveyed the occurrence of adverse events during the 20 weeks after ^131^I-mIBG injection and reported all severe adverse events in detail. We performed both examinations and imaging diagnosis 12 weeks after ^131^I-mIBG injection to evaluate the therapeutic effect. We administered the next course on finding neither severe adverse reactions nor progression of the disease during the previous course.

### Ethical considerations and registration

We conducted this study in accordance with the International Committee for Harmonization Good Clinical Practice (ICH—GCP) guideline and the Declaration of Helsinki. The institutional review boards of all participating institutions (Kanazawa University ethics committee, Gunma University ethics committee, Kagoshima University ethics committee, and Hokkaido University ethics committee) approved the study protocol. All patients provided informed consent before registration. This study was registered with UMIN Clinical Trials Registry (UMIN000018497) and the date of registration was July 30, 2015.

### Collaborative institutions

Currently, four institutions have the facilities to perform ^131^I-mIBG therapy in Japan in patients with refractory PPGL. This study was conducted at all these institutions—Kanazawa University Hospital, Gunma University Hospital, Hokkaido University Hospital, and Kagoshima University Hospital. Each of these institutions had at least one physician of nuclear medicine certified by the Japanese Society of Nuclear Medicine.

### Endpoint

In this study, our primary endpoint was DLT. Toxicities were graded according to the National Cancer Institute Common Terminology Criteria for Adverse Events (CTCAE) version 4.0. We defined DLT as follows: grade ≥ 4, hematological toxicity; grade ≥ 3, non-hematological toxicity except for grade 3 nausea, vomiting, anorexia, and hypertension. We evaluated DLT during the first 12 weeks after the first ^131^I-mIBG injection.

Our secondary endpoints were response rates according to RECIST 1.1^29^ and scintigraphic evaluation of ^123^I-mIBG, OS, PFS, and adverse event/reaction.

### Response evaluation

We performed primary response assessment at 20 weeks after enrollment. We graded the overall best responses based on computed tomography (CT) and ^123^I-mIBG scintigraphy.

We graded the CT response using the RECIST for measurable soft tissue disease and the sum of longest diameters (sLD) measured at study entry. For target lesions, we defined CR as the disappearance of all target lesions and reduction in the short axis of any pathological lymph node to <10 mm. We defined PR as a decrease of ≥30% in the sLD, SD as change that did not meet the definition of PR or PD, and PD as an increase of at least 20%, with the sum also demonstrating an absolute increase of at least 5 mm in the sLD or at a new site. For non-target lesions, we defined CR as the disappearance of all target lesions, with all lymph nodes of a non-pathological size with a short axis of <10 mm and the normalization of serum and urine catecholamine values. We interpreted residual radiographic abnormality as representing either fibrosis or scarring. We defined non-CR/non-PD as the persistence of one or more non-target lesions and serum and urine catecholamine values greater than the upper limit of the normal value. We defined PD as the unequivocal progression of existing non-target lesions. When, for any reason, examination was not performed, we defined the patient with the target or non-target lesions as NE. We performed comprehensive evaluation based on RECIST (Table 4). After the ^131^I-mIBG therapy, we evaluated the best overall response criteria based on RECIST (Table 5).

**Table 4:** Comprehensive evaluation based on RECIST.

Target lesion	Non-target lesion	New lesion	CE
CR	CR	No	CR
CR	non-CR/non-PD	No	PR
CR	NE	No	PR
PR	non-PD or NE	No	PR
SD	non-PD or NE	No	SD
NE	Non-PD	No	NE
PD	—	—	PD
—	PD	—	PD
—	—	Yes	PD
—	CR	No	CR
—	non-CR/non-PD	No	non-CR/non-PD
—	NE	No	NE
—	PD	—	PD
—	—	Yes	PD

CE: comprehensive evaluation; RECIST: Response Evaluation Criteria in Solid Tumours; CR: complete response; PR: partial response; NE: not evaluated; PD: progression disease; SD: stable disease.

**Table 5:** RECIST best overall response criteria.

CE			BOR
1st mIBG therapy	2nd mIBG therapy	3rd mIBG therapy	
CR or PR	SD	PD	SD
CR or PR	SD	NE	SD
CR or PR	PD	—	PD
CR or PR	NE	NE	NE
CR or PR	NE	SD	SD
SD	PD	—	PD
SD	SD	PD	SD
SD	NE	PD	PD
NE	NE	PD	PD
NE	NE	NE	NE
NE	NE	PD	PD

CE: comprehensive evaluation; BOR: best overall response; RECIST: Response Evaluation Criteria in Solid Tumours; mIBG: meta-iodobenzylguanidine; CR: complete response; PR: partial response; NE: not evaluated; SD: stable disease; PD: progressive disease.

We evaluated the scintigraphic response for target and non-target lesions, with target lesions meeting the following criteria: 1) a lesion must be confirmed clearly on ^123^I-mIBG whole body scintigraphy; (2) a lesion must be confirmed clearly on ^123^I-mIBG single photon emission tomography (SPECT); (3) a lesion must be observed on CT; and (4) a background region must be set on the SPECT image. We also recorded other non-target lesions. We measured the increased rate of mean count (IR_mean_) and maximum count (IR_max_) on target lesions using the following method: IR = (count in target region − count in background region)/count in background lesion. We defined background regions as follows: (1) liver—if a target lesion was in the liver and liver uptake was normal; (2) ipsilateral area—if a lesion was not in the midline and ipsilateral uptake was normal; (3) thigh—if a lesion was not in the thigh and abnormal uptake was -not observed; and (4) intracranial cavity—if a lesion was not in the intracranial cavity and abnormal uptake was not observed. We calculated the reduction rates (RRs) of IR_max_ and IR_mean_ (RR_max_ and RR_mean_) using the following method:

$${\rm{RR}}=({\rm{IR}}\,{\rm{at}}\,{\rm{baseline}}-{\rm{IR}}\,{{\rm{after}}}^{131}{\rm{I}} \mbox{-} {\rm{mIBG}}\,{\rm{therapy}})/{\rm{IR}}\,{\rm{at}}\,{\rm{baseline}}$$

For target lesions, we defined CR as the disappearance of all target lesions, where RR_max_ and RR_mean_ were >90%. We defined PR as a decrease of ≥30% in RR_max_ and RR_mean_, where RR_max_ and RR_mean_ were at least >90% in one lesion. We defined SD as change that did not meet the definition of PR or PD and PD as an increase of at least 30% in RR_max_ or RR_mean_ in one target lesion. For non-target lesions, we defined CR as the disappearance of all non-target lesions and catecholamine values within normal limits. We defined PD as the significant progression of non-target lesions, including relapse. We defined non-CR/non-PD as change that did not meet the definition of CR or PD. When, for any reason, an examination was not performed, we defined the patient with the target or non-target lesions as NE. We performed comprehensive evaluation based on ^123^I-mIBG scintigraphy after ^131^I-mIBG therapy (Table 6).

**Table 6:** Comprehensive evaluation based on ^123^I-mIBG scintigraphy.

Target lesion	Non-target lesion	New lesion	CE
CR	CR	No	CR
CR	non-CR/non-PD	No	PR
CR	NE	No	PR
PR	non-CR/non-PD or NE	No	PR
SD	non-CR/non-PD or NE	No	SD
NE	Non-PD	No	NE
PD	—	—	PD
—	PD	—	PD
—	—	Yes	PD
—	CR	No	CR
—	non-CR/non-PD	No	non-CR/non-PD
—	NE	No	NE
—	PD	—	PD
—	—	Yes	PD

mIBG: meta-iodobenzylguanidine; CR: complete response; PR: partial response; NE: not evaluated; CE: comprehensive evaluation; SD: stable disease; PD: progression disease.

### Eligibility criteria

Prior to enrollment in the study, patients must fulfill all of the following criteria: confirmed diagnosis of refractory PPGLs; no prior history of surgical treatment or radical external irradiation; aged ≥ 20 years; having an Eastern Cooperative Oncology Group Performance Status of 0–2 or a Karnofsky Performance Scale of ≥80%; independence in feeding, excretion, and sleeping; written informed consent; and having adequate bone marrow, liver, renal, and respiratory function, as shown below.

(i) white blood cell ≥ 3,000/mm^3^
(ii) hemoglobin ≥ 9.0 g/dL
(iii) platelets ≥ 100,000/mm^3^ without G-CSF
(iv) estimated glomerular filtration rate ≥ 30 mL/min/1.73 m^2^
(v) aspartate transaminase < 100 IU/L
(vi) alanine transaminase < 100 IU/L
(vii) lactate dehydrogenase < 400 IU/L
(viii) New York Heart Association Functional Classification class I or below
(ix) HbA1c < 8.0%
(x) oxygen saturation ≥ 96% at room air

In this study, we defined refractory PPGL as follows: PPGLs with severe local invasion at initial diagnosis; PPGLs with metastasis at initial diagnosis; PPGLs with local recurrence after surgical resection; and malignant PPGLs with metastasis after surgical resection^8^.

### Exclusion criteria

Patients were excluded for any of the following reasons: having had malignancies of other histologies apart from thyroid medullary carcinoma with multiple endocrine neoplasia type 2, angioblastoma of the retina with von Hippel–Lindau disease, and neurofibroma with neurofibromatosis type 1 within the preceding 5 years; having a history of tumor deterioration, CTCAE grade ≥ 2 non-hematologic toxicity, or grade ≥ 3 hematologic toxicity while undergoing ^131^I-mIBG therapy; having any CTCAE grade ≥ 2 toxicity; having a verified hepatitis B or C virus antibody or human immunodeficiency virus antibody positivity; having any other infections currently being treated; having a history of episodes of severe symptoms due to uncontrollable increase of catecholamines, severe arrhythmia, or asystole; having a diagnosis of uncontrollable symptomatic arrhythmia, thyroid dysfunction (hyperthyroidism or hypothyroidism), respiratory disease, or pleural effusion or ascites; having a diagnosis of coronary artery disease, amiodarone-treated arrhythmia, severe valvular disease of the heart, aortic disease, bleeding disorder, or psychosis; being a pregnant or lactating woman or a woman planning to become pregnant; having a diagnosis for any diseases currently treated with adrenal corticosteroids or immunosuppressants; no ability to stay in an isolated room for radiation control; having episodes of allergic reaction to potassium iodide; or having any symptomatic lesions currently treated with palliative external irradiation.

### Patient registration

We sent a patient registration form to the independent data center of an academic research organization at Kanazawa University Hospital. We ran patient registration between February 1, 2016 and July 31, 2017. We continued making observations until October 31, 2017.

### Treatment

We planned a treatment protocol in accordance with the Japanese draft guidelines for ^131^I-mIBG therapy by the Drafting Committee for Guidelines of Radiotherapy with ^131^I-mIBG, Committee for Nuclear Oncology and Immunology, Japanese Society of Nuclear Medicine, and referred to the procedure guidelines for ^131^I-mIBG therapy by the European Association of Nuclear Medicine^30,31^.

After admitting the patients to an isolated radiation treatment room, we administered 7.4 GBq of ^131^I-mIBG (ATC Code: V10X A02, National Centre for Nuclear Research Radioisotope Centre POLATOM, Poland) injection over 1 h at day 0. Because the maximum permitted amount of radioisotope agents in Hokkaido University Hospital is 5.55 GBq, one patient received 5.55 GBq of ^131^I-mIBG. Before and after injection, we noted blood pressure, heart rate, and the presence of any symptoms. We discharged the patients from the radiation treatment room when they had satisfied the release criteria set out by Japanese regulations.

### Prescribed, recommended, or acceptable supportive treatments

Oral administration of potassium iodide was prescribed for the protection of the thyroid gland and 5-hydroxytryptamine (serotonin) receptor antagonist was recommended to avoid vomiting, whereas bisphosphonates and denosumab were acceptable for coadministration.

### Second or third ^131^I-mIBG therapy

For patients who had not experienced severe adverse reactions or progression of disease in 20 weeks after the first course, we administered the second and third course of ^131^I-mIBG therapy every 24 weeks until the end of study period.

### Follow-up schedule

The study period extended from the date of enrollment to 20 weeks after the ^131^I-mIBG injections. We collected the results of physical and blood examinations at enrollment every day from day 0 to day 4 and 2, 4, 6, 8, 12, 16, and 20 weeks after the ^131^I-mIBG injection to evaluate its safety. We evaluated its efficacy by making comparisons between baseline and 12 weeks after ^131^I-mIBG injection. We performed physiological examinations, electrocardiography, cardiac ultrasonography, blood examination (common and catecholamine), urinary examination, CT, and ^123^I-mIBG scintigraphy on the day of enrollment and 12 weeks after ^131^I-mIBG injection.

### Sample size

Our target sample size was a total of 20 patients, based on the precision of a one-sided 90% CI estimate of the DLT rate. More specifically, the upper confidence limit using the exact method in 15 evaluable patients and 2 observed DLTs (13%) would rule out a null rate of 33%. It is commonly considered acceptable in chemotherapy using a cytotoxic agent that DLT can occur in one-third or less of patients. Therefore, the incidence of the DLT would be allowed if it occurred in two or fewer patients under ^131^I-mIBG therapy. Because of the limited use of radioactive drugs, each of our institute could only perform ^131^I-mIBG therapy on a limited number of patients. In considering this problem, we determined the feasible number of treated patients to be 15. We determined the total number of patients for registration allowing for a drop-out rate of approximately 20%.

### Statistical analysis

We included all treated patients in the population analyzed for the primary endpoint. We summarized the DLT using the occurrence rate and calculated the CI of the DLT rate using an exact method based on binomial distribution.

Our secondary endpoints included PFS, OS, and toxicity. We used the date of the ^131^I-mIBG injection as the starting point for the Kaplan–Meier estimation of PFS and OS. For PFS, we defined an event as PD or death from any cause. For a simple summary of the outcome by group, we based the PFS and OS rates on Kaplan–Meier estimates. All reported P values are two-sided.

## Supplementary information

Study protocol

## References

[1] Wieland DM, Swanson DP, Brown LE, Beierwaltes WH (1979). Imaging the adrenal medulla with an I-131-labeled antiadrenergic agent. J Nucl Med.

[2] Bomanji J (1987). Uptake of iodine-123 MIBG by pheochromocytomas, paragangliomas, and neuroblastomas: a histopathological comparison. J Nucl Med.

[3] Vaidyanathan G (2008). Meta-iodobenzylguanidine and analogues: chemistry and biology. Q J Nucl Med Mol Imaging.

[4] Taieb D (2012). EANM 2012 guidelines for radionuclide imaging of phaeochromocytoma and paraganglioma. Eur J Nucl Med Mol Imaging.

[5] Castellani MR, Chiti A, Seregni E, Bombardieri E (2000). Role of 131I-metaiodobenzylguanidine (MIBG) in the treatment of neuroendocrine tumours. Experience of the National Cancer Institute of Milan. Q J Nucl Med.

[6] Lenders JW, Eisenhofer G, Mannelli M, Pacak K (2005). Phaeochromocytoma. Lancet.

[7] Kimura N (2014). Pathological grading for predicting metastasis in phaeochromocytoma and paraganglioma. Endocr Relat Cancer.

[8] Lam AK (2017). Update on Adrenal Tumours in 2017 World Health Organization (WHO) of Endocrine Tumours. Endocr Pathol.

[9] Fitzgerald PA (2006). Malignant pheochromocytomas and paragangliomas: a phase II study of therapy with high-dose 131I-metaiodobenzylguanidine (131I-MIBG). Ann N Y Acad Sci.

[10] Gonias S (2009). Phase II study of high-dose [131I]metaiodobenzylguanidine therapy for patients with metastatic pheochromocytoma and paraganglioma. J Clin Oncol.

[11] Noto RB (2018). Phase 1 Study of High-Specific-Activity I-131 MIBG for Metastatic and/or Recurrent Pheochromocytoma or Paraganglioma. J Clin Endocrinol Metab.

[12] Inaki A (2017). A phase I clinical trial for [(131)I]meta-iodobenzylguanidine therapy in patients with refractory pheochromocytoma and paraganglioma: a study protocol. J Med Invest.

[13] van Hulsteijn LT, Niemeijer ND, Dekkers OM, Corssmit EP (2014). (131)I-MIBG therapy for malignant paraganglioma and phaeochromocytoma: systematic review and meta-analysis. Clin Endocrinol (Oxf).

[14] Loh KC, Fitzgerald PA, Matthay KK, Yeo PP, Price DC (1997). The treatment of malignant pheochromocytoma with iodine-131 metaiodobenzylguanidine (131I-MIBG): a comprehensive review of 116 reported patients. J Endocrinol Invest.

[15] Yoshinaga K (2014). Effects and safety of (1)(3)(1)I-metaiodobenzylguanidine (MIBG) radiotherapy in malignant neuroendocrine tumors: results from a multicenter observational registry. Endocr J.

[16] Gedik GK, Hoefnagel CA, Bais E, Olmos RA (2008). 131I-MIBG therapy in metastatic phaeochromocytoma and paraganglioma. Eur J Nucl Med Mol Imaging.

[17] Ezziddin S (2012). Repeated Radionuclide therapy in metastatic paraganglioma leading to the highest reported cumulative activity of 131I-MIBG. Radiat Oncol.

[18] Baudin E (2014). Therapy of endocrine disease: treatment of malignant pheochromocytoma and paraganglioma. Eur J Endocrinol.

[19] Eisenhofer G (2004). Malignant pheochromocytoma: current status and initiatives for future progress. Endocr Relat Cancer.

[20] Fiebrich HB (2009). 6-[F-18]Fluoro-L-dihydroxyphenylalanine positron emission tomography is superior to conventional imaging with (123)I-metaiodobenzylguanidine scintigraphy, computer tomography, and magnetic resonance imaging in localizing tumors causing catecholamine excess. J Clin Endocrinol Metab.

[21] Rufini V, Treglia G, Perotti G, Giordano A (2013). The evolution in the use of MIBG scintigraphy in pheochromocytomas and paragangliomas. Hormones (Athens).

[22] Safford, S. D. *et al*. Iodine -131 metaiodobenzylguanidine is an effective treatment for malignant pheochromocytoma and paraganglioma. *Surgery* **134**, 956–962; discussion 962–953, 10.1016/S0039 (2003). 10.1016/s0039-6060(03)00426-4 14668728

[23] Tan TH, Hussein Z, Saad FF, Shuaib IL (2015). Diagnostic Performance of (68)Ga-DOTATATE PET/CT, (18)F-FDG PET/CT and (131)I-MIBG Scintigraphy in Mapping Metastatic Pheochromocytoma and Paraganglioma. Nucl Med Mol Imaging.

[24] Fonte JS (2012). False-negative 123I-MIBG SPECT is most commonly found in SDHB-related pheochromocytoma or paraganglioma with high frequency to develop metastatic disease. Endocr Relat Cancer.

[25] Sisson JC, Shulkin BL, Esfandiari NH (2006). Courses of malignant pheochromocytoma: implications for therapy. Ann N Y Acad Sci.

[26] Wakabayashi H (2013). Prognostic values of initial responses to low-dose (131)I-MIBG therapy in patients with malignant pheochromocytoma and paraganglioma. Ann Nucl Med.

[27] Averbuch SD (1988). Malignant pheochromocytoma: effective treatment with a combination of cyclophosphamide, vincristine, and dacarbazine. Ann Intern Med.

[28] Hescot S (2013). One-year progression-free survival of therapy-naive patients with malignant pheochromocytoma and paraganglioma. J Clin Endocrinol Metab.

[29] Eisenhauer EA (2009). New response evaluation criteria in solid tumours: revised RECIST guideline (version 1.1). Eur J Cancer.

[30] Giammarile F (2008). EANM procedure guidelines for 131I-meta-iodobenzylguanidine (131I-mIBG) therapy. Eur J Nucl Med Mol Imaging.

[31] Kinuya S (2015). Draft guidelines regarding appropriate use of I - MIBG radiotherapy for neuroendocrine tumors: Guideline Drafting Committee for Radiotherapy with I - MIBG, Commit- tee for Nuclear Oncology and Immunology, The Japanese Society of Nuclear Medicine. Ann Nucl Med.

