# Author Correction: A phase I clinical trial for [^131^I]meta-iodobenzylguanidine therapy in patients with refractory pheochromocytoma and paraganglioma

**DOI:** 10.1038/s41598-022-05239-2

**Published:** 2022-01-19

**Authors:** Hiroshi Wakabayashi, Anri Inaki, Kenichi Yoshimura, Toshinori Murayama, Yasuhito Imai, Tetsuya Higuchi, Megumi Jinguji, Tohru Shiga, Seigo Kinuya

**Affiliations:** 1 Department of Nuclear Medicine, Kanazawa University Hospital, Ishikawa, Japan; 2 Department of Biostatistics, Innovative Clinical Research Center, Kanazawa University Hospital, Ishikawa, Japan; 3 Department of Clinical Development, Kanazawa University Hospital, Ishikawa, Japan; 4 Department of Data Center, Innovative Clinical Research Center, Kanazawa University Hospital, Ishikawa, Japan; 5 Department of Diagnostic Radiology and Nuclear Medicine, Gunma University Graduate School of Medicine, Gunma, Japan; 6 Department of Radiology, Kagoshima University Graduate School of Medical and Dental Sciences, Kagoshima, Japan; 7 Department of Nuclear Medicine, Hokkaido University, Hokkaido, Japan

Correction to: *Scientific Reports* 10.1038/s41598-019-43880-6, published online 20 May 2019

The original version of this article contained an error in the 95% confidence intervals calculated for the second and third courses of ^131^I-mIBG.

As a result, in the Results section under the subheading ‘Response evaluation’,

“Eight patients received a second course of ^131^I-mIBG therapy. The scintigraphic response in the second course was 5% in CR (1/20), 10% in PR (2/20), 20% in SD (4/20), 5% in PD (1/20), and 60% in unknown (12/20). The response rate was 15% (95% CI: 6.0–61.0%).”

now reads:

“Eight patients received a second course of ^131^I-mIBG therapy. The scintigraphic response in the second course was 5% in CR (1/20), 10% in PR (2/20), 20% in SD (4/20), 5% in PD (1/20), and 60% in unknown (12/20). The response rate was 15% (95% CI: 3.2–37.9%).”

“Three patients received a third course of ^131^I-mIBG therapy. There were three patients with PR (15%) and 17 patients with unknown (85%) in the scintigraphic response. The response rate was 15% (95% CI: 29.2–100.0%).”

now reads:

“Three patients received a third course of ^131^I-mIBG therapy. There were three patients with PR (15%) and 17 patients with unknown (85%) in the scintigraphic response. The response rate was 15% (95% CI: 3.2–37.9%).”

The original Article has been corrected.

