# Supplementary material for: A phase I clinical trial for [131I]meta-iodobenzylguanidine therapy in patients with refractory pheochromocytoma and paraganglioma
Source: Sci Rep. 2019 May 20;9:7625. doi: 10.1038/s41598-019-43880-6 (PMC6527850; doi:10.1038/s41598-019-43880-6)
Supplement: Supplementary file 1 — Study protocol [file 41598_2019_43880_MOESM1_ESM.pdf]

ORIGINAL

A phase I clinical trial for [^{131}I]meta-iodobenzylguanidine therapy in patients with refractory pheochromocytoma and paraganglioma : a study protocol

Anri Inaki[1], Kenichi Yoshimura[2], Toshinori Murayama[3], Yasuhito Imai[4], Yoshikazu Kuribayashi[5], Tetsuya Higuchi[6], Megumi Jinguji[7], Tohru Shiga[8], and Seigo Kinuya[1]

[1]*Department of Nuclear Medicine, Faculty of Medicine, Institute of Medical, Pharmaceutical and Health Sciences, Kanazawa University, Ishikawa, Japan,* [2]*Department of Biostatistics, Innovative Clinical Research Center, Kanazawa University Hospital, Ishikawa Japan,* [3]*Department of Clinical Development, Kanazawa University Hospital, Ishikawa, Japan,* [4]*Department of Data Center, Innovative Clinical Research Center, Kanazawa University Hospital, Ishikawa, Japan,* [5]*Department of Monitoring and Auditing in Clinical Trials, Innovative Clinical Research Center, Kanazawa University Hospital, Ishikawa, Japan* [6]*Department of Diagnostic Radiology and Nuclear Medicine, Gunma University Graduate School of Medicine, Gunma, Japam* [7]*Department of Radiology, Kagoshima University Graduate School of Medical and Dental Sciences, Kagoshima, Japan* [8]*Department of Nuclear Medicine, Hokkaido University, Hokkaido, Japan*

Abstract : ***Objective*** **Pheochromocytoma and paraganglioma (PPGLs) are rare neuroendocrine tumors derived from the adrenal medulla or extra-adrenal paraganglioma from extra-adrenal chromaffin tissue. Although malignant PPGLs has miserable prognosis, the treatment strategy remains to be established. An internal radiation therapy using [^{131}I]meta-iodobenzylguanidine (^{131}I-mIBG) called MIBG therapy has been attempted as one of the systemic treatment of malignant PPGLs. The aim of this study is therefore to evaluate the safety and the efficacy of MIBG therapy for refractory PPGLs.** ***Methods*** **Patients with refractory PPGLs will be enrolled in this study. The total number of patients for registration is 20. The patients receive a fixed dose of 7,400 MBq of ^{131}I-mIBG. Adverse events are surveyed during 20 weeks after ^{131}I-mIBG injection and all severe adverse events will be documented and reported in detail in accordance with the Common Terminology Criteria for Adverse Events (CTCAE). Examination and imaging diagnosis are performed in 12 weeks after ^{131}I-mIBG injection for the evaluation of therapeutic effect in accordance with the Response Evaluation in Solid Tumours (RECIST).** ***Conclusion*** **The current study is the first multi-institutional prospective study of MIBG therapy and thereby will play a significant role in improving the patients' prognosis of refractory PPGLs. J. Med. Invest. 64 : 205-209, August, 2017**

Keywords **:** *Pheochromocytoma, Paraganglioma, ^{131}I-mIBG, Prospective study protocol*

MAIN DOCUMENT

Background

Pheochromocytoma and paraganglioma (PPGLs) are rare tumors genealogically derived from neural crest cells which develop sympathetic and parasympathetic nervous system. The World Health Organization (WHO) classification defines pheochromocytoma as the tumor arising from the adrenal medulla and extra-adrenal paraganglioma from extra-adrenal chromaffin tissue. Malignant PPGLs are commonly defined as the tumor with metastatic lesion. The incidence of PPGLs is 2 to 8 per million persons per year and malignant PPGLs occur in 10% to 30% of all PPGLs cases (1, 2). The gold standard of treatment strategy for non-malignant PPGLs is surgical resection and the 5-year survival rate is estimated at approximately 90% (3). However, around 10% of the patients with successful surgical resection develop a recurrence and 50% of them have distant metastasis (4, 5). Once distant metastasis at initial diagnosis or re-staging is detected, the 5-year survival rate is reduced to 40% to 50% (6-8). Although the treatment strategy for malignant PPGLs remains to be established, some systemic treatments have been attempted. Among the systemic treatments, chemotherapy using cyclophosphamide, vincristine and dacarbazine called CVD therapy and internal radiation therapy using [^{131}I]meta-iodobenzylguanidine (^{131}I-mIBG) called MIBG therapy have been attempted so widely that meta-analyses of these treatments were reported. The meta-analysis of CVD therapy showed that the rate of complete response, partial response and stable disease on imaging were 4%, 37% and 14%, respectively (9).

^{131}I-mIBG is a radioactive agent with high-energy beta-ray emission first developed by Wieland and Sisson et al. in 1979 (10). It is specifically taken up through its neuronal uptake-1 receptor to tumor cells derived from such neural crest cells as pheochromocytoma, medullary thyroid cancer and neuroblastoma, which leads to anticancer effect to these tumors. Therefore, MIBG therapy has been attempted to treat such neuroendocrine tumors as PPGLs, medullary thyroid carcinoma and neuroblastoma (11-13). For malignant PPGLs, a meta-analysis of retrospective studies reported that the response rate on imaging and in hormonal examination was 0-83% and 20-100%, respectively (14). However, no prospective studies have been performed yet because of the extremely low incidence of malignant PPGLs.

In accordance with these studies, our institutes have performed MIBG therapy since early 21th century and 5 institutes including us can perform MIBG therapy in Japan at present. In this result, on the Advanced Medical Care Committee sponsored by Japanese Ministry of Health, Labour and Welfare held on November 2013, ^{131}I-mIBG was promoted to an anticancer drug which should be developed with high medical needs. Therefore, MIBG therapy for the patients with PPGLs was authorized as one of the Japanese Advanced Medical Care Program B with high medical needs.

Received for publication February 28, 2017 ; accepted March 20, 2017.

Address correspondence and reprint requests to Anri Inaki Department of Nuclear Medicine, Faculty of Medicine, Institute of Medical, Pharmaceutical and Health Sciences, Kanazawa University, 13-1 Takaramachi, Kanazawa, Japan and Fax : +81-76-234-4257.

Therefore, we aim in this study to establish the protocol of prospective clinical trial of MIBG therapy ahead of sponsor initiated clinical trial and to demonstrate the safety and the efficacy of MIBG therapy for the patients with refractory PPGLs.

METHODS AND DESIGN

Study outline

The diagram of the study process is shown in Figure1. Patients with refractory PPGLs are enrolled in this study. After screened in accordance with both inclusion and exclusion criteria, the registered patients receive a fixed dose of 7,400 MBq of ^{131}I-mIBG. The occurrence of adverse events is surveyed during 20 weeks after ^{131}I-mIBG injection and all severe adverse events will be documented and reported in detail. Both examination and imaging diagnosis are performed in 12 weeks after ^{131}I-mIBG injection for the evaluation of therapeutic effect. The next course will be performed when neither severe adverse reactions nor progression of disease is found in the previous course.

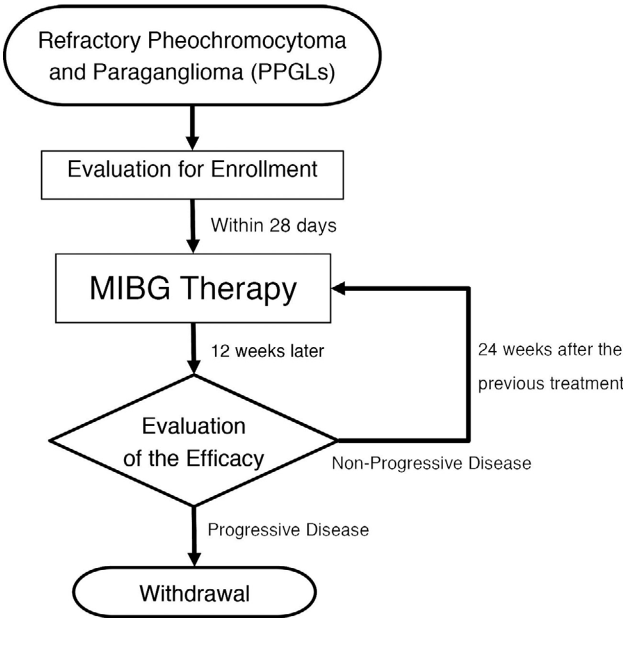

Figure 1.
Study Procedure of this study.

Purpose

The primary objective is to evaluate the safety and the efficacy of MIBG therapy for refractory PPGLs which have no conventional treatments.

Study design

This study is an open-label, multi-institutional single arm clinical trial, in which participating institutions include 4 specialized centers in Japan of January 2017. Participating institutions are listed in Appendix 1.

Ethical considerations and registration

This study is conducted in accordance with the International Committee for Harmonization Good Clinical Practice (ICH-GCP) guideline and the Declaration of Helsinki. The study protocol was approved by the institutional review board of all participating institutions. Informed consent will be provided for all patients before registration. This study was registered with UMIN clinical Trials Registry (UMIN000018497).

Endpoint

The primary endpoint of this study is dose limiting toxicity (DLT). Toxicities will be graded according to the National Cancer Institute Common Terminology Criteria for Adverse Events (CTCAE) version 4.0. DLT is defined as follows ; grade ≥ 4 hematological toxicity ; grade ≥ 3 non-hematological toxicity except for grade 3 nausea, vomiting, anorexia and hypertension. DLT will be evaluated during first 12 weeks (84 days) after first mIBG injection.

The secondary endpoints are response rate by RECIST and by scintigraphic evaluation of MIBG, overall survival, progression-free survival, and adverse event/reaction.

Eligibility criteria

Inclusion criteria

Prior to enrollment in the study, patients must fulfill all of the following criteria : confirmed refractory PPGLs, which includes ^{123}I-mIBG-avid pheochromocytoma, paraganglioma, malignant pheochromocytoma, and malignant paraganglioma ; no prior history of surgical treatment and radical external irradiation ; Age 20 years or older ; Eastern Cooperative Oncology Group Performance Status of 0 to 2 or Karnofsky Performance Scale $\geq$80% ; independent feeding, excretion and sleeping ; written informed consent ; having adequate bone marrow, liver, renal, and respiratory function as shown below.

i) WBC $\geq$3,000/mm3.
ii) Hb $\geq$9.0 g/dL
iii) Platelets $\geq$100,000/mm3 without G-CSF.
iv) eGFR $\geq$30 mL/min/1.73 m2.
v) AST $<$100 IU/L.
vi) ALT $<$100 IU/L.
vii) LDH $<$400 IU/L.
viii) New York Heart Association (NYHA) Functional Classification class I or below.
ix) HbA1c $<$8.0%
x) Oxygen saturation $\geq$96[%] at room air.

In this study, refractory PPGLs are defined as follows ; PPGLs with severe local invasion at initial diagnosis, malignant PPGLs with metastasis at initial diagnosis, PPGLs with local recurrence after surgical resection and malignant PPGLs with metastasis after surgical resection.

Exclusion criteria

Patients will be excluded for any of the following reasons : having malignancies of other histologies within 5 years except for thyroid medullary carcinoma with multiple endocrine neoplasia type 2, angioblastoma of retina with von Hippel-Lindau disease and neurofibroma with neurofibromatosis type 1 ; having history of tumor deterioration, CTCAE grade $\geq$2 non-hematologic toxicity, or grade $\geq$3 hematologic toxicity under the condition of MIBG therapy ; having any CTCAE grade $\geq$2 toxicity ; verified hepatitis B virus antigen or C virus antibody, or human immunodeficiency virus antibody positivity ; having any other infections currently treated ; episodes of severe symptoms due to uncontrollable increase of catecholamines, fatal arrhythmia or asystole ; diagnosed as uncontrollable symptomatic arrhythmia, thyroid dysfunction (hyperthyroidism or hypothyroidism), respiratory disease, or

pleural effusion or ascites ; diagnosed as coronary artery disease, amiodarone-treated arrhythmia, severe valvular disease of the heart, aortic disease, bleeding disorder, or psychosis ; Pregnant or lactating women, or women who were planning to become pregnant ; diagnosed as any diseases currently treated with adrenal corticosteroids or immunosuppressants ; not applicable isolation due to radiation control ; having episodes of allergic reaction to potassium iodide ; having any symptomatic lesions currently treated with palliative external irradiation.

Patient registration

The investigators send a patient registration form to the independent data center at an academic research organization at Kanazawa University Hospital. Patient registration began on February 1st 2016 and is to continue until July 31th, 2017.

Treatment

A treatment protocol was planned in accordance with the Japanese draft guidelines of MIBG therapy by Drafting Committee for Guideline of Radiotherapy with ^{131}I-MIBG, Committee for Nuclear Oncology and Immunology, the Japanese Society of Nuclear Medicine (JSNM) and referred to the procedure guidelines for ^{131}I-mIBG therapy by the European Association of Nuclear Medicine (EANM) (15, 16).

After admission to an isolated radiation treatment room, the patients are received 7,400 MBq of ^{131}I-mIBG injection over 1 hour at day 0. If the permitted amount of radioisotope agents in each institute is lower than 7,400 MBq, patients are received maximal dose of permitted amount of radioisotopes. Before and after injection, blood pressure, heart rate and the presence of any symptoms are remarked on. The patients will be discharged from the radiation treatment room when satisfying the release criteria regulated by the Japanese regulation.

Prescribed, recommended or acceptable supportive treatments

Oral administration of potassium iodide should be performed for the protection of the thyroid gland and 5-HT3 receptor antagonist, bisphosphonates and denosumab are acceptable for coadministration.

Second or third MIBG therapy

For patients who have not experienced severe adverse reactions or progression of disease in 24 weeks after the previous course, the second and the third course of MIBG therapy will be performed.

Follow-up schedule

The follow-up schedule for evaluating the safety and the efficacy is shown in Table 1. The study period will be from date of enrollment to 20 weeks after ^{131}I-mIBG injection. Data to evaluate the safety of this study will be collected at enrollment, baseline, every day from day 0 to day 4 and 2, 4, 6, 8, 12, 16 and 20 weeks after ^{131}I-mIBG injection. The efficacy of this study will also be evaluated with the comparison between baseline and 12 weeks after ^{131}I-mIBG injection.

The independent committee for evaluating safety and efficacy is instituted and would be called if unexpected severe adverse reactions would occur. All severe adverse events including death of any reasons, unexpected admission and unexpected prolonged admission shall be immediately reported to Japanese Ministry of Health, Labour and Welfare.

Sample size

Target sample size was a total of 20 patients. Sample size was based on precision of a one-sided 90% confidence interval (CI) estimate of DLT rate. More specifically, in 15 evaluable patients and 2 DLTs (13%) observed, the upper confidence bound using the exact method would rule out a null rate of 33%. For chemotherapy using cytotoxic agent, it is commonly considered acceptable that DLT can occur in one third or less of patients. Therefore, the incidence of the DLT would be allowed if the DLT would occur in 2 or less patients under MIBG treatment. On account of the limited use of radioactive drug, each of our institute can perform MIBG therapy to less than a certain number of patients. Considering this problem, the feasible number of treated patients was 15. Moreover, allowing for a drop-out rate of approximately 20%, the total number of patients for registration is determined.

Statistical analysis

The population analyzed for the primary endpoint included the

Table 1. Follow-up schedule

	Enrollment	Treatment		Observation						
Clinical assessments, testing and investigations	Day of enrollment	within 7 days before treatment	Day 0 to Day 4	Day 14	Day 28	Day 42	Day 56	Day 84	Day 112	Day 140
Physical examinations	+	+	+	+	+	+	+	+	+	+
Electrocardiogram	+							+		
Cardiac ultrasound	+							+		
Blood examination (common)	+	+		+	+	+	+	+	+	+
Blood examination (catecholamines)	+							+		
Urinary examination	+							+		
Computed tomography	+							+		
^{123}I-mIBG scintigraphy	+							+		

all treated patients The DLT is summarized using the rate of occurrence. The CI of DLT rate is calculated using exact method based on binomial distribution.

RESULTS AND DISCUSSION

This is the first multi-institutional prospective study in Japan to evaluate the safety and the efficacy of MIBG therapy for ^{123}I-mIBG-avid refractory PPGLs, along with the standardized treatment protocol and the pre-specified follow-up schedule. Although some therapeutic strategies against malignant PPGLs have been suggested, prospective and systematic treatment protocols, let alone randomized controlled trials, were not established because of the extremely low incidence of PPGLs. Our study will play a significant role as a breakthrough of this problem in improving the prognosis of the patients with malignant PPGLs. Additionally, this study was performed in accordance with the Japanese Advanced Medical Care Program B ahead of sponsor initiated registration trial, which intend to rationalize application for approval in accordance with the Japanese Pharmaceutical and Medical Device Act.

In this study, we define the tumor satisfying these 2 conditions above as "refractory" PPGLs and eligible for this study. Although PPGLs with local invasion beyond surgical operation is commonly not included in malignant PPGLs according to diagnostic criteria, its clinical features and prognosis are considered to be similar to malignant PPGLs owing to incapability of curative treatment.

Previous retrospective studies revealed that the incidence of grade 3 or higher adverse reactions was quite low and grade 4 hematological toxicity had never occurred in the fixed dose of 7,400 MBq of ^{131}I-mIBG (13, 17-22). However no prospective study for the evaluation of adverse reactions has been reported and therefore it is considered to be needed in order to demonstrate the safety of MIBG therapy.

DLT is defined as follows ; grade $\geq$4 hematological toxicity ; grade $\geq$3 non-hematological toxicity except for grade 3 nausea, vomiting, anorexia and hypertension. We excluded nausea from non-hematological toxicity. Considering the therapeutic effect to malignant tumor, nausea was one of the common adverse reactions in this therapy and therefore grade 3 nausea was concluded to be allowed. And furthermore, grade 3 of vomiting, anorexia and hypertension are also defined as acceptable on account of the same reason.

CONFLICT OF INTEREST

The current study has been supported by a grant from the Japan Agency for Medical Research and Development (AMED).

ACKNOWLEDGEMENT

The current work has been supported by a grant from the Japan Agency for Medical Research and Development (AMED).

REFERENCES

1. Lenders JW, Eisenhofer G, Mannelli M, Pacak K : Phaeochromocytoma. Lancet 366(9486) : 665-75, 2005. doi : 10.1016/S 0140-6736(05)67139-5. PubMed PMID : 16112304.
2. Kimura N, Takayanagi R, Takizawa N, Itagaki E, Katabami T, Kakoi N, et al : Pathological grading for predicting metastasis in phaeochromocytoma and paraganglioma. Endocrine-related cancer 21(3) : 405-14, 2014. doi : 10.1530/ERC-13-0494. PubMed PMID : 24521857.
3. Plouin PF, Chatellier G, Fofol I, Corvol P : Tumor recurrence and hypertension persistence after successful pheochromocytoma operation. Hypertension 29(5) : 1133-9, 1997. PubMed PMID : 9149678.
4. van Heerden JA, Roland CF, Carney JA, Sheps SG, Grant CS : Long-term evaluation following resection of apparently benign pheochromocytoma(s)/paraganglioma(s). World journal of surgery 14(3) : 325-9, 1990. PubMed PMID : 1973322.
5. Amar L, Servais A, Gimenez-Roqueplo AP, Zinzindohoue F, Chatellier G, Plouin PF : Year of diagnosis, features at presentation, and risk of recurrence in patients with pheochromocytoma or secreting paraganglioma. The Journal of clinical endocrinology and metabolism 90(4) : 2110-6, 2005. doi : 10.1210/jc.2004-1398. PubMed PMID : 15644401.
6. Averbuch SD, Steakley CS, Young RC, Gelmann EP, Goldstein DS, Stull R, et al : Malignant pheochromocytoma : effective treatment with a combination of cyclophosphamide, vincristine, and dacarbazine. Annals of internal medicine 109(4) : 267-73, 1988. PubMed PMID : 3395037.
7. Eisenhofer G, Bornstein SR, Brouwers FM, Cheung NK, Dahia PL, de Krijger RR, et al : Malignant pheochromocytoma : current status and initiatives for future progress. Endocrine-related cancer 11(3) : 423-36, 2004. PubMed PMID : 15369446.
8. Hescot S, Leboulleux S, Amar L, Vezzosi D, Borget I, Bournaud-Salinas C, et al : One-year progression-free survival of therapy-naive patients with malignant pheochromocytoma and paraganglioma. The Journal of clinical endocrinology and metabolism 98(10) : 4006-12, 2013. doi : 10.1210/jc.2013-1907. PubMed PMID : 23884775.
9. Niemeijer ND, Alblas G, van Hulsteijn LT, Dekkers OM, Corssmit EP : Chemotherapy with cyclophosphamide, vincristine and dacarbazine for malignant paraganglioma and pheochromocytoma : systematic review and meta-analysis. Clinical endocrinology 81(5) : 642-51, 2014. doi : 10.1111/cen.12542. PubMed PMID : 25041164.
10. Wieland DM, Swanson DP, Brown LE, Beierwaltes WH : Imaging the adrenal medulla with an I-131-labeled antiadrenergic agent. J Nucl Med 20(2) : 155-8, 1979. PubMed PMID : 430189.
11. Shapiro B : A review of the status of radio-iodinated-MIBG therapy for neuroendocrine tumors. Int Med 2 : 61-8, 1991
12. Castellani MR, Chiti A, Seregni E, Bombardieri E : Role of 131I-metaiodobenzylguanidine (MIBG) in the treatment of neuroendocrine tumours. Experience of the National Cancer Institute of Milan. The quarterly journal of nuclear medicine : official publication of the Italian Association of Nuclear Medicine 44(1) : 77-87, 2000. PubMed PMID : 10932604.
13. Safford SD, Coleman RE, Gockerman JP, Moore J, Feldman JM, Leight GS, Jr., et al : Iodine-131 metaiodobenzylguanidine is an effective treatment for malignant pheochromocytoma and paraganglioma. Surgery 134(6) : 956-62, 2003. discussion 62-3. doi : 10.1016/S0039. PubMed PMID : 14668728.
14. van Hulsteijn LT, Niemeijer ND, Dekkers OM, Corssmit EP : (131)I-MIBG therapy for malignant paraganglioma and phaeochromocytoma : systematic review and meta-analysis. Clinical endocrinology 80(4) : 487-501, 2014. doi : 10.1111/cen.12341. PubMed PMID : 24118038.
15. Kinuya S, Yoshinaga K, Higuchi T, Jinguji M, Kurihara H, Kawamoto H : Draft guidelines regarding appropriate use of I-MIBG radiotherapy for neuroendocrine tumors : Guideline Drafting Committee for Radiotherapy with I-MIBG, Committee for Nuclear Oncology and Immunology, The Japanese Society of Nuclear Medicine. Ann Nucl Med : 2015. doi : 10.1007/s12149-015-0960-z. PubMed PMID : 25773397.
16. Giammarile F, Chiti A, Lassmann M, Brans B, Flux G, Eanm :

EANM procedure guidelines for 131I-meta-iodobenzylguanidine (131I-mIBG) therapy. Eur J Nucl Med Mol Imaging 35(5) : 1039-47, 2008. doi : 10.1007/s00259-008-0715-3. PubMed PMID : 18274745.
17. Loh KC, Fitzgerald PA, Matthay KK, Yeo PP, Price DC : The treatment of malignant pheochromocytoma with iodine-131 metaiodobenzylguanidine (131I-MIBG) : a comprehensive review of 116 reported patients. Journal of endocrinological investigation 20(11) : 648-58, 1997. PubMed PMID : 9492103.
18. Mukherjee JJ, Kaltsas GA, Islam N, Plowman PN, Foley R, Hikmat J, et al : Treatment of metastatic carcinoid tumours, phaeochromocytoma, paraganglioma and medullary carcinoma of the thyroid with (131)I-meta-iodobenzylguanidine [(131)I-mIBG]. Clinical endocrinology 55(1) : 47-60, 2001. PubMed PMID : 11453952.
19. Gedik GK, Hoefnagel CA, Bais E, Olmos RA : 131I-MIBG therapy in metastatic phaeochromocytoma and paraganglioma. Eur J Nucl Med Mol Imaging 35(4) : 725-33, 2008. doi : 10.1007/s00259-007-0652-6. PubMed PMID : 18071700.
20. Castellani MR, Seghezzi S, Chiesa C, Aliberti GL, Maccauro M, Seregni E, et al : (131)I-MIBG treatment of pheochromocytoma : low versus intermediate activity regimens of therapy. The quarterly journal of nuclear medicine and molecular imaging : official publication of the Italian Association of Nuclear Medicine 54(1) : 100-13, 2010. PubMed PMID : 20168292.
21. Yoshinaga K, Oriuchi N, Wakabayashi H, Tomiyama Y, Jinguji M, Higuchi T, et al : Effects and safety of I-metaiodobenzylguanidine (MIBG) radiotherapy in malignant neuroendocrine tumors : Results from a multicenter observational registry. Endocrine journal : 2014. PubMed PMID : 25214026.
22. Wakabayashi H, Taki J, Inaki A, Nakamura A, Kayano D, Fukuoka M, et al : Prognostic values of initial responses to low-dose (131)I-MIBG therapy in patients with malignant pheochromocytoma and paraganglioma. Ann Nucl Med 27(9) : 839-46, 2013. doi : 10.1007/s12149-013-0755-z. PubMed PMID : 23864328.
